# Association between stress hyperglycemic ratio and all-cause mortality in critically ill patients following cardiac surgery: A retrospective cohort study

**DOI:** 10.1097/MD.0000000000050418

**Published:** 2026-08-28

**Authors:** Yuanli Yang, Ronglin Chen, Jian Xu, Sanming Yu, Linhai Sun, Ying Zhao

**Affiliations:** a Department of Critical Care Medicine, Longgang Central Hospital (Shenzhen Clinical College, Guangzhou University of Chinese Medicine; Longgang Clinical Institute of Shantou University Medical College), Shenzhen, Guangdong, China; b Department of Emergency Medicine, Longgang Central Hospital (Shenzhen Clinical College, Guangzhou University of Chinese Medicine; Longgang Clinical Institute of Shantou University Medical College), Shenzhen, Guangdong, China.

**Keywords:** all-cause mortality, cardiac surgery, cohort study, predictive, stress hyperglycemia ratio

## Abstract

The stress hyperglycemic ratio (SHR) can be used as a reliable predictor of various adverse cardiovascular outcomes. However, there are fewer studies on the relationship between SHR and the risk of all-cause mortality in critically ill patients undergoing cardiac surgery (CS). Therefore, the aim of this study was to investigate the relationship between SHR levels and all-cause mortality in critically ill patients with CS admitted to the intensive care unit. Data from 3117 CS intensive care unit patients (Medical Information Mart for Intensive Care-IV) were analyzed. SHR values were categorized into 4 groups using equal-frequency binning to ensure uniform distribution across intervals. The primary outcomes were 30- and 180-day all-cause mortality, with secondary outcomes including 90- and 365-day all-cause mortality. Kaplan–Meier survival curves were employed to compare survival outcomes across SHR groups for both primary and secondary endpoints. To further evaluate the associations between SHR and mortality outcomes, Cox proportional hazards regression models were fitted, supplemented by restricted cubic splines analysis to investigate potential nonlinear relationships between SHR and the risk of death. Among 3117 participants, higher levels of the SHR index were significantly associated with increased all-cause mortality risk at 30, 90, 180, and 365 days (log-rank *P* < .001 by Kaplan–Meier analysis). Cox proportional hazards regression confirmed significantly elevated mortality risk in the highest SHR quartile at all timepoints. restricted cubic splines analysis revealed a J-shaped association between SHR and mortality, with inflection points at 0.94 (30-day) and 0.96 (365--day). Compared to patients with SHR levels below the inflection point, those with higher SHR levels had a 4.490-fold higher risk of 30-day all-cause mortality (hazard ratio: 4.490, 95% confidence interval: 2.944–6.848) and 3.701-fold higher risk of 365-day all-cause mortality (hazard ratio: 3.701, 95% confidence interval: 2.526–5.425). In critically ill patients following CS, higher SHR was significantly associated with increased all-cause mortality risk at 30, 90, 180 and 365 days postoperatively. SHR may serve as a prognostic marker to guide intensive care unit therapeutic strategies.

## 1. Introduction

The American Heart Association documents that millions of patients require cardiac surgical interventions annually.^[1]^ While refinements in operative methodologies and perioperative care protocols have substantially improved survival rates, mortality remains clinically consequential, with approximately 2% of patients succumbing even after low-risk procedures such as coronary artery bypass grafting or aortic valve replacement.^[2]^ This persistent risk profile imposes multidimensional burdens, adversely affecting patients’ families and straining healthcare infrastructure. Notably, mortality escalates disproportionately in cases involving complex surgical interventions or high-risk patient cohorts.^[3,4]^ These observations collectively emphasize the critical importance of precise prognostic evaluation in contemporary cardiac surgery (CS) practice.

Stress hyperglycemia ratio (SHR) constitutes a maladaptive pathophysiological response characterized by acute glucose elevation secondary to endogenous or exogenous stressors.^[5]^ Although admission blood glucose has been widely adopted as a surrogate marker for stress hyperglycemia, its diagnostic precision in discriminating acute glycemic surges is compromised by preexisting chronic dysglycemia.^[6,7]^ The SHR metric, calculated through normalization of admission blood glucose to glycated hemoglobin (HbA1c) levels, eliminates baseline glycemic confounders, thereby enabling precise quantification of stress-induced hyperglycemic magnitude.^[8]^ Contemporary clinical evidence substantiates that SHR reliably captures dynamic stress-mediated glucose dysregulation patterns during hospitalization.^[9]^

Although stress hyperglycemia may provide metabolic support under certain conditions, in critically ill patients this hyperglycemic state is often strongly associated with poor clinical outcomes. Previous studies have shown independent associations between stress hyperglycemia and adverse outcomes in patients with sepsis,^[10]^ acute heart failure,^[11]^ acute myocardial infarction,^[12]^ and atrial fibrillation.^[13]^ However, evidence regarding SHR specifically in CS patients, particularly those with critical illness, remains limited. Therefore, the aim of this study was to investigate the association between SHR and short- and long‑term mortality in this population, with the goal of evaluating its potential as a prognostic biomarker.

## 2. Methods

### 2.1. Data sources

In this retrospective cohort study, data were sourced from Medical Information Mart for Intensive Care-IV (MIMIC-IV) version 3.1, a publicly available critical care database curated by the Computational Physiology Laboratory. This dataset includes de-identified clinical records of over 190,000 patients. It covers 450,000 inpatient admissions at Beth Israel Deaconess Medical Center between 2008 and 2022. The BIDMC Institutional Review Board granted ethical approval for this retrospective analysis with waiver of informed consent under institutional anonymized data use protocols. Patient inclusion was strictly based on CS diagnostic codes from International Classification of Diseases, 9th and 10th Revision codes. Certified database access was obtained by Principal Investigator Ying Zhao through institutional authorization (certificate ID: 66372905).

### 2.2. Inclusion and exclusion criteria

Inclusion criteria: age 18 to 90 years and patients who underwent cardiac surgical procedures.

Exclusion criteria: intensive care unit (ICU) length of stay <24 hours; missing admission laboratory values of initial blood glucose and HbA1c; and exclusion of subsequent ICU admissions for patients with multiple hospitalizations, retaining only the index admission data. Demographics: age, sex, marital status, and body mass index. Physiological parameters: temperature, heart rate, systolic/diastolic blood pressure, respiratory rate, and oxygen saturation. Initial ICU laboratory profiles: blood glucose, leukocyte count, hemoglobin level, platelet count, urinary nitrogen, and serum creatinine. Comorbidities: hypertension, diabetes mellitus types 1/2, chronic obstructive pulmonary disease, chronic kidney disease, myocardial infarction, and stroke. Severity assessment: acute physiology score III, sequential organ failure assessment score, simplified acute physiology score II. SHR was calculated using: SHR = admission blood glucose (mg/dL)/(28.7 × HbA1c [%] − 46.7).^[8]^

### 2.3. Study size and bias

This analysis included all eligible patients from the MIMIC-IV database; no prior sample size calculation was performed. We minimized selection bias through prespecified inclusion criteria and addressed confounding through multivariable adjustment.

### 2.4. Outcome measures

The primary outcome measures were 30- and 180-day all-cause mortality, while the secondary outcome measures included 90- and 365-day all-cause mortality.

### 2.5. Statistical methods

Missing data exceeding 20% were excluded, while variables with ≤20% missingness underwent multiple imputation. Multicollinearity was addressed by eliminating variables with variance inflation factors >5. Continuous and categorical variables were respectively expressed as mean ± standard deviation and counts (percentages) and were analyzed using χ^2^ or Fisher exact tests. SHR was stratified into quartiles by equal-frequency binning. Survival differences across SHR groups were evaluated using Kaplan–Meier curves with log-rank testing, and restricted cubic spline models were used to detect nonlinear associations. Cox proportional hazards regression was performed to quantify mortality relationships, with subgroup analyses conducted across age, sex, myocardial infarction, diabetes, and cancer cohorts. All analyses employed Decision Linnc 1.0 (Hangzhou Yuantong Information Technology Co., Ltd), with statistical significance defined as 2-tailed *P* < .05. All core statistical analyses were performed using R version 4.3.1 (with packages including survival, rms, and glmnet).

## 3. Results

### 3.1. Baseline data

**Figure 1.**
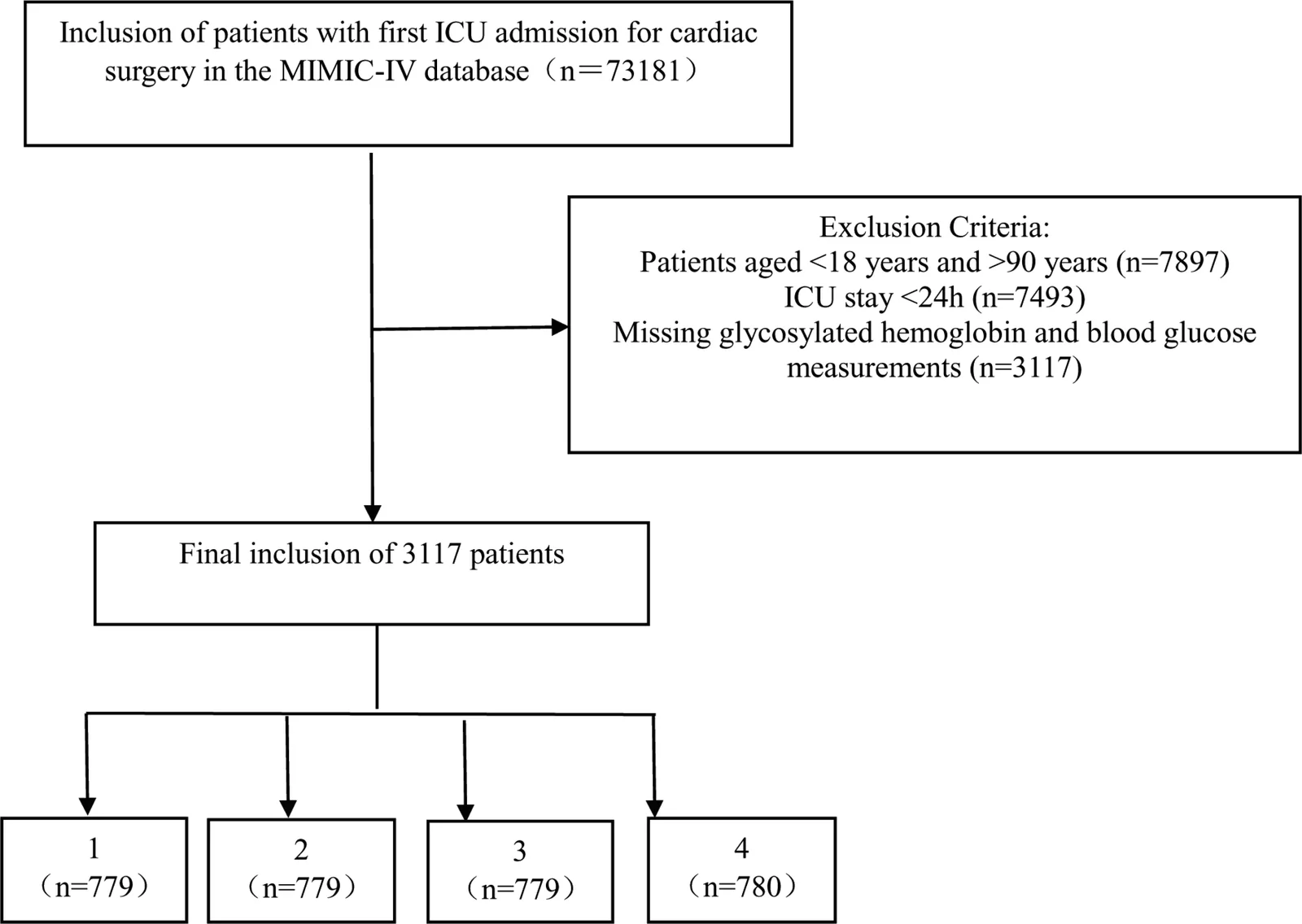
Flow chart of this study. ICU = intensive care unit, MIMIC = Medical Information Mart for Intensive Care.

Data from 3117 CS ICU patients were extracted from MIMIC-IV (Fig. 1). Table 1 presents the baseline characteristics of the study population. Among them, 2188 (70.20%) were male, 1673 (53.67%) had hypertension, 1049 (33.65%) had type 2 diabetes, 33 (1.06%) had type 1 diabetes, 991 (31.79%) had heart failure, 431 (13.83%) had malignant tumors, 250 (8.02%) had a history of stroke, 393 (12.61%) had pneumonia, 633 (20.31%) had acute renal failure, and 1987 (63.75%) had hyperlipidemia. Patients were stratified into quartiles based on SHR: quartile 1 (0.27 ≤ SHR < 0.80), quartile 2 (0.80 ≤ SHR < 0.97), quartile 3 (0.97 ≤ SHR < 1.15), and quartile 4 (1.16 ≤ SHR ≤ 1.69), with 779 patients in each of the 1st 3 quartiles and 780 patients in the 4th quartile. Patients in quartile 4 exhibited higher sequential organ failure assessment scores, acute physiology score III scores, simplified acute physiology score II scores, heart rate, diastolic blood pressure, respiratory rate, white blood cell count, anion gap, international normalized ratio, blood urea nitrogen, and serum creatinine, as well as lower oxygen saturation, serum chloride, pH, and partial pressure of arterial oxygen (Table 1).

**Table 1:** Baseline characteristics of studied population.

Variable	Overall, N = 3117	*T*1 (0.27–0.80), N = 779	*T*2 (0.80–0.97), N = 779	*T*3 (0.97–1.15), N = 779	*T*4 (1.16–1.69), N = 780	*P*
Age (yr)	68.06 ± 11.52	68.85 ± 10.66	68.16 ± 11.67	67.92 ± 11.9	67.33 ± 11.78	.07
BMI (kg/m^2^)	29.87 ± 5.81	30.45 ± 5.64	30.06 ± 5.92	29.61 ± 5.78	29.33 ± 5.85	<.01
SOFA score	5.34 ± 2.82	5.44 ± 2.72	5.26 ± 2.8	5.02 ± 2.65	5.65 ± 3.06	<.01
APS III score	39.64 ± 18.46	40.93 ± 18.71	39.72 ± 19.15	36.82 ± 16.14	41.08 ± 19.36	<.01
SAPS II score	36.04 ± 12.09	36.87 ± 12.17	36.22 ± 12.49	34.8 ± 11.31	36.27 ± 12.3	.01
HR (beats/min)	81.28 ± 12.98	80.58 ± 11.49	80.96 ± 12.79	80.65 ± 12.42	82.95 ± 14.86	<.01
SBP (mm Hg)	112.4 ± 18.77	112.53 ± 18.31	112.59 ± 18.94	112.35 ± 18.81	112.12 ± 19.04	.96
DBP (mm Hg)	61.64 ± 13.22	60.57 ± 12.71	61.22 ± 13.23	62.16 ± 12.85	62.58 ± 13.98	.01
RR (time/min)	15.54 ± 6.21	15.07 ± 4.68	15.27 ± 5.76	15.55 ± 8.03	16.25 ± 5.85	<.01
SpO_2_ (%)	98.59 ± 2.68	98.96 ± 2.16	98.64 ± 2.31	98.61 ± 2.4	98.16 ± 3.54	<.01
Temperature (°C)	97.81 ± 3.63	97.62 ± 5.02	97.87 ± 2.38	97.97 ± 2.48	97.79 ± 3.94	.27
Hematocrit	29.51 ± 5.77	28.48 ± 5.63	29.69 ± 5.74	30.11 ± 5.75	29.77 ± 5.85	<.01
Hemoglobin	9.84 ± 2.02	9.47 ± 1.98	9.91 ± 2.01	10.06 ± 2.01	9.92 ± 2.04	<.01
PLT (10^9^/L)	160.49 ± 67.39	162.95 ± 62.57	162.69 ± 74.26	160.34 ± 64.49	155.96 ± 67.55	.14
RDW	13.99 ± 1.55	14.09 ± 1.48	14.06 ± 1.56	13.81 ± 1.48	13.99 ± 1.64	<.01
RBC (10^9^/L)	3.26 ± 0.67	3.19 ± 0.67	3.29 ± 0.67	3.31 ± 0.68	3.26 ± 0.67	<.01
WBC (10^9^/L)	12.76 ± 5.55	12.37 ± 5.33	12.44 ± 5.36	12.73 ± 5.19	13.49 ± 6.2	<.01
Anion gap (mmol/L)	11.51 ± 3.26	11.19 ± 2.89	11.34 ± 3.06	11.45 ± 3.07	12.07 ± 3.87	<.01
Total calcium (mmol/L)	8.31 ± 0.59	8.35 ± 0.6	8.31 ± 0.56	8.3 ± 0.55	8.28 ± 0.65	.15
Chloride	107.49 ± 4.54	107.91 ± 4.17	107.67 ± 4.43	107.44 ± 4.41	106.93 ± 5.06	<.01
Potassium	4.32 ± 0.57	4.34 ± 0.55	4.32 ± 0.57	4.3 ± 0.54	4.34 ± 0.62	.53
Sodium (mmol/L)	138.18 ± 3.21	138.43 ± 2.77	138.36 ± 3.09	137.98 ± 3.21	137.95 ± 3.69	<.01
Creatinine	1.12 ± 1.06	1.12 ± 0.99	1.09 ± 0.97	1.03 ± 0.83	1.22 ± 1.37	<.01
BUN (mg/dL)	19.12 ± 11.62	19.53 ± 11.2	18.81 ± 11.01	18.04 ± 10.15	20.08 ± 13.71	<.01
INR	1.47 ± 0.49	1.43 ± 0.28	1.45 ± 0.37	1.5 ± 0.73	1.52 ± 0.48	<.01
Lactate (mmol/L)	2.09 ± 1.1	2.15 ± 1.06	2.04 ± 1.03	2 ± 0.94	2.15 ± 1.32	.01
PaCO_2_ (mm Hg)	40.78 ± 6.79	40.53 ± 6.45	40.83 ± 7.09	40.58 ± 6.25	41.2 ± 7.33	.19
pH	7.4 ± 0.07	7.41 ± 0.06	7.4 ± 0.07	7.4 ± 0.06	7.39 ± 0.07	<.01
PaO_2_ (mm Hg)	308.58 ± 110.99	315.64 ± 103.27	314.12 ± 108.12	312.57 ± 108.29	292 ± 121.93	<.01
Gender						.03
Male	2188 (70.20%)	516 (66.24%)	547 (70.22%)	563 (72.27%)	562 (72.05%)	
Female	929 (29.80%)	263 (33.76%)	232 (29.78%)	216 (27.73%)	218 (27.95%)	
Acute kidney injury						<.01
No	2484 (79.69%)	629 (80.74%)	642 (82.41%)	643 (82.54%)	570 (73.08%)	
Yes	633 (20.31%)	150 (19.26%)	137 (17.59%)	136 (17.46%)	210 (26.92%)	
Pneumonia						.07
No	2724 (87.39%)	667 (85.62%)	684 (87.80%)	702 (90.12%)	671 (86.03%)	
Yes	393 (12.61%)	112 (14.38%)	95 (12.20%)	77 (9.88%)	109 (13.97%)	
Cerebrovascular accident						.14
No	2867 (91.98%)	709 (91.01%)	723 (92.81%)	729 (93.58%)	706 (90.51%)	
Yes	250 (8.02%)	70 (8.99%)	56 (7.19%)	50 (6.42%)	74 (9.49%)	
Diabetes 2						<.01
No	2068 (66.35%)	261 (33.50%)	554 (71.12%)	628 (80.62%)	625 (80.13%)	
Yes	1049 (33.65%)	518 (66.50%)	225 (28.88%)	151 (19.38%)	155 (19.87%)	
Diabetes 1						<.01
No	3084 (98.94%)	762 (97.82%)	772 (99.10%)	777 (99.74%)	773 (99.10%)	
Yes	33 (1.06%)	17 (2.18%)	7 (0.90%)	2 (0.26%)	7 (0.90%)	
Hyperlipidemia						<.01
No	1130 (36.25%)	232 (29.78%)	273 (35.04%)	292 (37.48%)	333 (42.69%)	
Yes	1987 (63.75%)	547 (70.22%)	506 (64.96%)	487 (62.52%)	447 (57.31%)	
Hypertension						<.01
No	1444 (46.33%)	344 (44.16%)	346 (44.42%)	345 (44.29%)	409 (52.44%)	
Yes	1673 (53.67%)	435 (55.84%)	433 (55.58%)	434 (55.71%)	371 (47.56%)	
Malignant tumor						.92
No	2686 (86.17%)	673 (86.39%)	663 (85.11%)	672 (86.26%)	678 (86.92%)	
Yes	431 (13.83%)	106 (13.61%)	116 (14.89%)	107 (13.74%)	102 (13.08%)	
Heart failure						.02
No	2126 (68.21%)	528 (67.78%)	538 (69.06%)	561 (72.02%)	499 (63.97%)	
Yes	991 (31.79%)	251 (32.22%)	241 (30.94%)	218 (27.98%)	281 (36.03%)	
360-d-mortality						<.01
No	3015 (96.73%)	752 (96.53%)	764 (98.07%)	762 (97.82%)	737 (94.49%)	
Yes	102 (3.27%)	27 (3.47%)	15 (1.93%)	17 (2.18%)	43 (5.51%)	
180-d-mortality						<.01
No	3026 (97.08%)	755 (96.92%)	768 (98.59%)	765 (98.20%)	738 (94.62%)	
Yes	91 (2.92%)	24 (3.08%)	11 (1.41%)	14 (1.80%)	42 (5.38%)	
90-d-mortality						<.01
No	3035 (97.37%)	757 (97.18%)	770 (98.84%)	765 (98.20%)	743 (95.26%)	
Yes	82 (2.63%)	22 (2.82%)	9 (1.16%)	14 (1.80%)	37 (4.74%)	
30-d-mortality						<.01
No	3054 (97.98%)	763 (97.95%)	772 (99.10%)	772 (99.10%)	747 (95.77%)	
Yes	63 (2.02%)	16 (2.05%)	7 (0.90%)	7 (0.90%)	33 (4.23%)	

APS II = acute physiology score II, BMI = body mass index, BUN = blood urea nitrogen, DBP = diastolic blood pressure, HR = hazard ratio, INR = international normalized ratio, PLT = platelet count, RBC = red blood cell, RDW = red blood cell distribution width, RR = respiratory rate, SAPS II = simplified acute physiology score II, SBP = systolic blood pressure, SHR = stress hyperglycemia ratio, SOFA = sequential organ failure assessment, SpO_2_ = pulse oximetry-derived oxygen saturation, WBC = white blood cell.

### 3.2. SHR and short-and long-term outcomes

**Figure 2.**
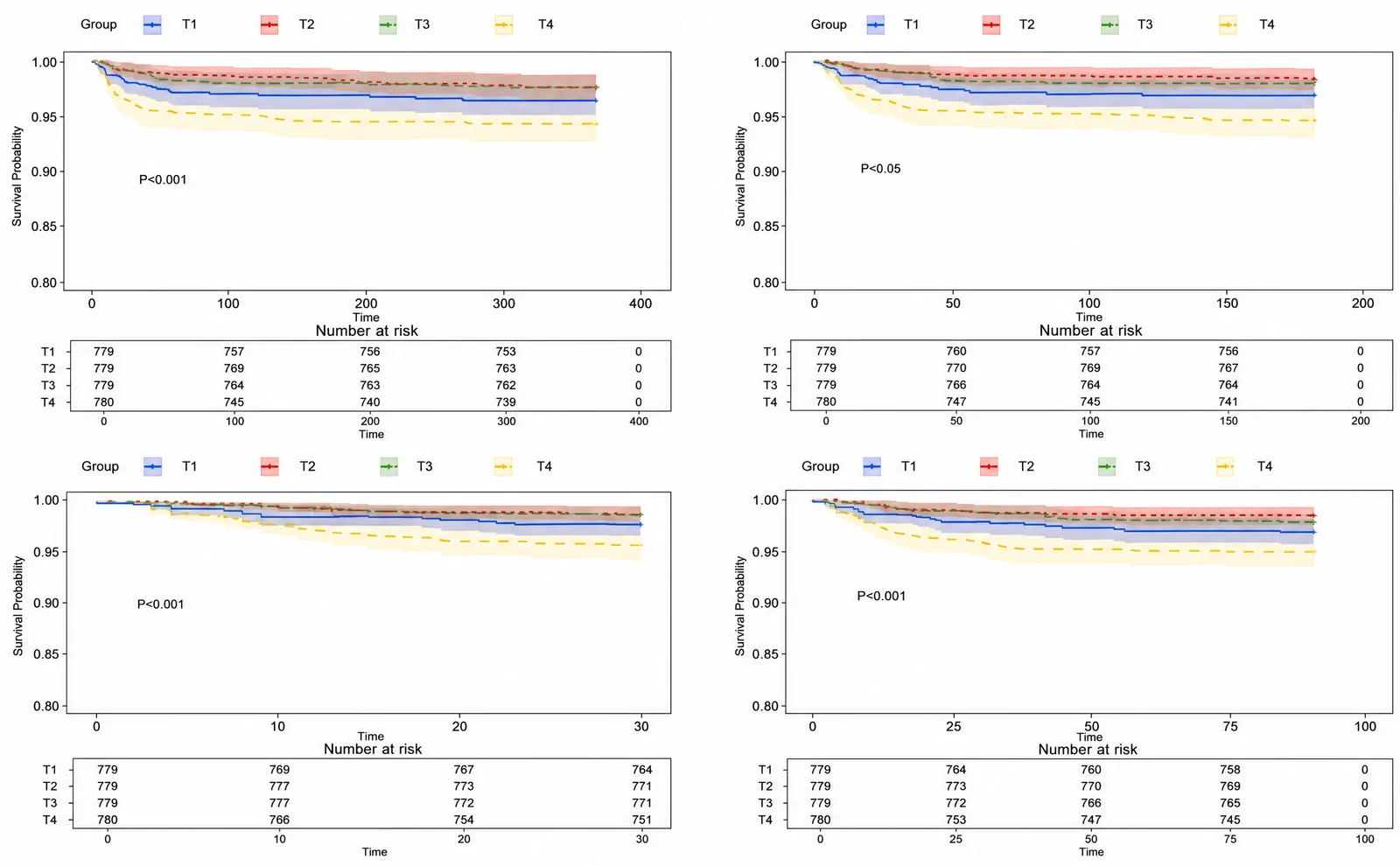
Kaplan–Meier survival curve. Quartile 1 (0.27 ≤ SHR < 0.80), quartile 2 (0.80 ≤ SHR < 0.97), quartile 3 (0.97 ≤ SHR < 1.15), and quartile 4 (1.15 ≤ SHR < 1.69). SHR = stress hyperglycemic ratio.

Kaplan–Meier analysis revealed significant differences in mortality at 30, 90, 180, and 365 days were observed across SHR groups. Quartile 4 (1.16 ≤ SHR ≤ 1.69) consistently had the highest all-cause mortality across all time points (Fig. 2).

**Figure 3.**
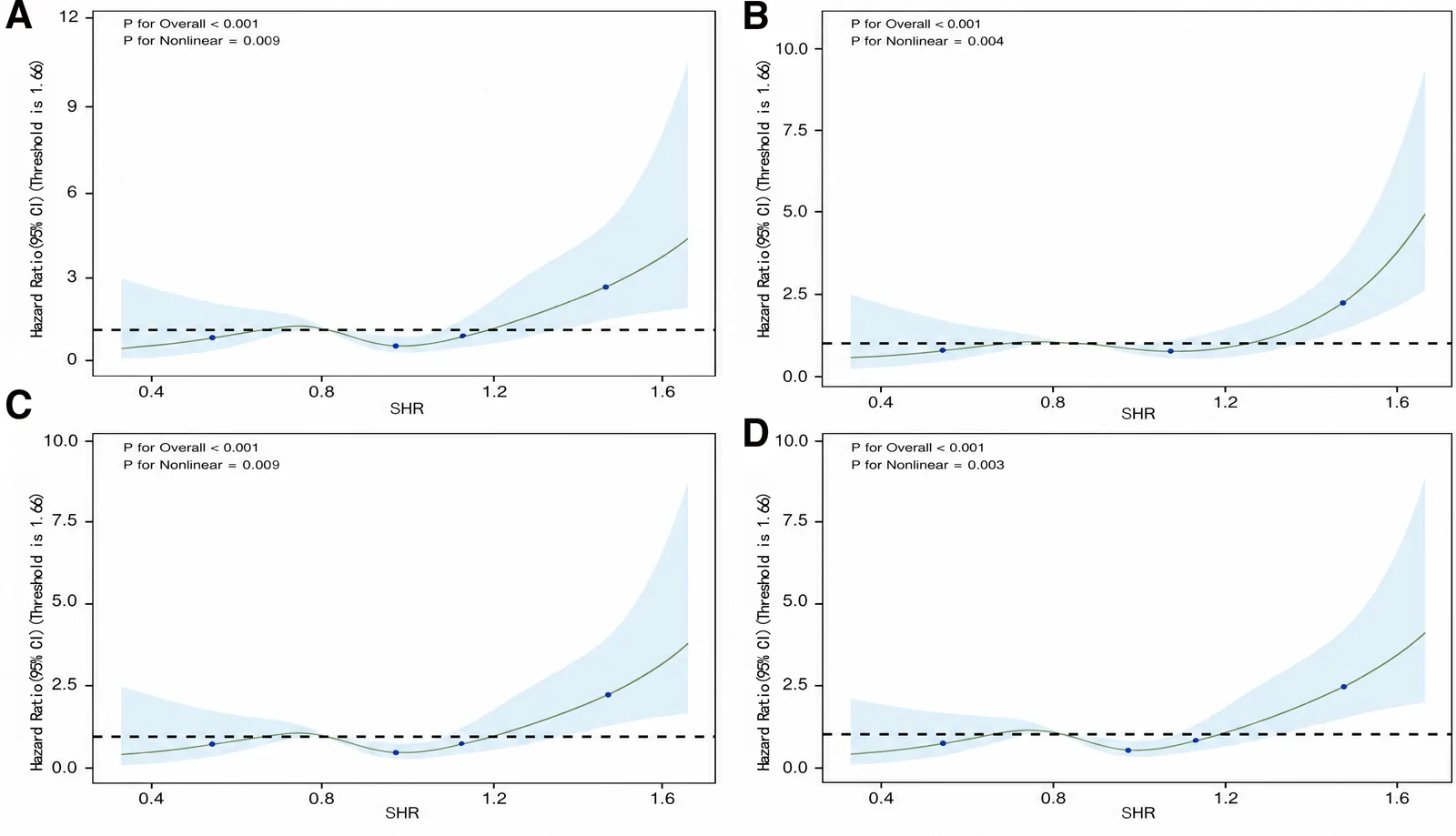
RCS curve for the SHR and 30-day: (A) 90-day, (B) 180-day, (C) 365-day, and (D) mortality. CI = confidence interval, RCS = restricted cubic splines, SHR = stress hyperglycemic ratio.

Furthermore, the restricted cubic splines analysis also revealed a “J-shaped” relationship between SHR and mortality at 30, 90, 180, and 365 days (*P* value for nonlinearity <.05 for all) (Fig. 3).

Cox proportional hazards models indicated that the highest quantile of the SHR (SHR, *Q*4) was an independent risk factor for all-cause mortality from short- to long-term follow-up. After adjustment for age, weight, height, sex, and multiple comorbidities, patients in *Q*4 had a significantly higher mortality risk compared to those in the lowest quantile (*Q*1) at 30 days (model 3: hazard ratio [HR] = 2.104, *P* = .030), 90 days (model 3: HR = 1.784, *P* = .049), 180 days (model 3: HR = 1.882, *P* = .025), and 365 days (model 3 HR = 1.616, *P* = .048) (Tables 2–5). In contrast, the middle SHR quantiles (*Q*2 and *Q*3) showed no statistically significant increase in risk at any follow-up time point (all *P* > .05).

**Table 2:** Cox regression model (30-day mortality).

Variables	Model 1		Model 2		Model 3	
	HR (95% CI)	*P*	HR (95% CI)	*P*	HR (95% CI)	*P*
SHR quantile						
1	Ref.		Ref.		Ref.	
2	0.53	.147	0.581	.227	0.595	.249
3	0.53	.147	0.641	.333	0.597	.272
4	1.948	.036	2.102	.029	2.104	.030

Model 2: Adjusted for: age, weight, height, sex, diabetes, hypertension, hyperlipidemia, malignancy.

Model 3: Adjusted for: age, weight, height, sex, diabetes, hypertension, hyperlipidemia, malignancy, WBC.

CI = confidence interval, HR = hazard ratio, SHR = stress hyperglycemia ratio.

**Table 3:** Cox regression model (90-day mortality).

Variables	Model 1		Model 2		Model 3	
	HR (95% CI)	*P*	HR (95% CI)	*P*	HR (95% CI)	*P*
SHR quantile						
1	Ref.		Ref.		Ref.	
2	0.451	.037	0.518	.093	0.528	.104
3	0.677	.244	0.829	.599	0.798	.536
4	1.607	.081	1.829	.039	1.784	.049

Model 2: Adjusted for: age, weight, height, sex, diabetes, hypertension, hyperlipidemia, malignancy.

Model 3: Adjusted for: age, weight, height, sex, diabetes, hypertension, hyperlipidemia, malignancy, WBC.

CI = confidence interval, HR = hazard ratio, SHR = stress hyperglycemia ratio.

**Table 4:** Cox regression model (180-day mortality)

Variables	Model 1		Model 2		Model 3	
	HR (95% CI)	*P*	HR(95% CI)	*P*	HR (95% CI)	*P*
SHR quantile						
1	Ref.		Ref.		Ref.	
2	0.560	.095	0.631	.198	0.638	.209
3	0.647	.190	0.780	.479	0.736	.392
4	1.758	.031	1.953	.017	1.882	.025

Model 2: Adjusted for age, weight, height, sex, diabetes, hypertension, hyperlipidemia, and malignancy.

Model 3: Adjusted for age, weight, height, sex, diabetes, hypertension, hyperlipidemia, malignancy, and WBC.

CI = confidence interval, HR = hazard ratio, SHR = stress hyperglycemia ratio.

**Table 5:** Cox regression model (365-day mortality).

Variables	Model 1		Model 2		Model 3	
	HR (95% CI)	*P*	HR (95% CI)	*P*	HR (95% CI)	*P*
SHR quantile						
1	Ref.		Ref.		Ref.	
2	0.648	.164	0.741	.294	0.710	.290
3	0.687	.221	0.824	.552	0.769	.429
4	1.596	.042	1.693	.049	1.616	.048

Model 2: Adjusted for age, weight, height, sex, diabetes, hypertension, hyperlipidemia, and malignancy.

Model 3: Adjusted for age, weight, height, sex, diabetes, hypertension, hyperlipidemia, malignancy, and WBC.

CI = confidence interval, HR = hazard ratio, SHR = stress hyperglycemia ratio.

To further investigate this nonlinear relationship, Cox proportional hazards models and 2-piece Cox proportional hazards models were employed (*P* < .05 for the log-likelihood ratio in both models) (Tables 6–9). The analyses identified inflection points at 0.94 for 30-day all-cause mortality and at 0.96 for mortality at other time points.

**Table 6:** Threshold effect analysis of SHR index on 30-day all-cause mortality in CS patients.

30-d mortality	HR (95% CI), *P* value
Fitting by the standard linear regression	3.781 (2.514–5.687), <.001
Fitting model by 2-piecewise linear regression	
Inflection point	0.94
SHR < 0.94	0.598 (0.080–4.451), .616
SHR ≥ 0.94	4.490 (2.944–6.848), <.001
*P* for log-likelihood ratio	.030

CI = confidence interval, CS = cardiac surgery, HR = hazard ratio, SHR = stress hyperglycemia ratio.

**Table 7:** Threshold effect analysis of SHR index on 90-day all-cause mortality in CS patients.

90-d mortality	HR (95% CI), *P* value
Fitting by the standard linear regression	3.153 (2.129–4.670), <.001
Fitting model by 2-piecewise linear regression	
Inflection point	0.94
SHR < 0.94	0.621 (0.115–3.347), .580
SHR ≥ 0.94	3.808 (2.534–5.723), <.001
*P* for log-likelihood ratio	.070

CI = confidence interval, CS = cardiac surgery, HR = hazard ratio, SHR = stress hyperglycemia ratio.

**Table 8:** Threshold effect analysis of SHR index on 180-day all-cause mortality in CS patients.

180-d mortality	HR (95% CI), *P* value
Fitting by the standard linear regression	3.119 (2.142–4.543), <.001
Fitting model by 2-piecewise linear regression	
Inflection point	0.94
SHR < 0.94	0.494 (0.104–2.339), .374
SHR ≥ 0.94	3.888 (2.647–5.709), <.001
*P* for log-likelihood ratio	.027

CI = confidence interval, CS = cardiac surgery, HR = hazard ratio, SHR = stress hyperglycemia ratio.

**Table 9:** Threshold effect analysis of SHR index on 365-day all-cause mortality in CS patients.

365-d mortality	HR (95% CI), *P* value
Fitting by the standard linear regression	2.910 (2.009–4.216), <.001
Fitting model by 2-piecewise linear regression	
Inflection point	0.96
SHR < 0.96	0.547 (0.137–2.184), .393
SHR ≥ 0.96	3.701 (2.526–5.425), <.001
*P* for log-likelihood ratio	.023

CI = confidence interval, CS = cardiac surgery, HR = hazard ratio, SHR = stress hyperglycemia ratio.

### 3.3. Subgroup analysis

**Figure 4.**
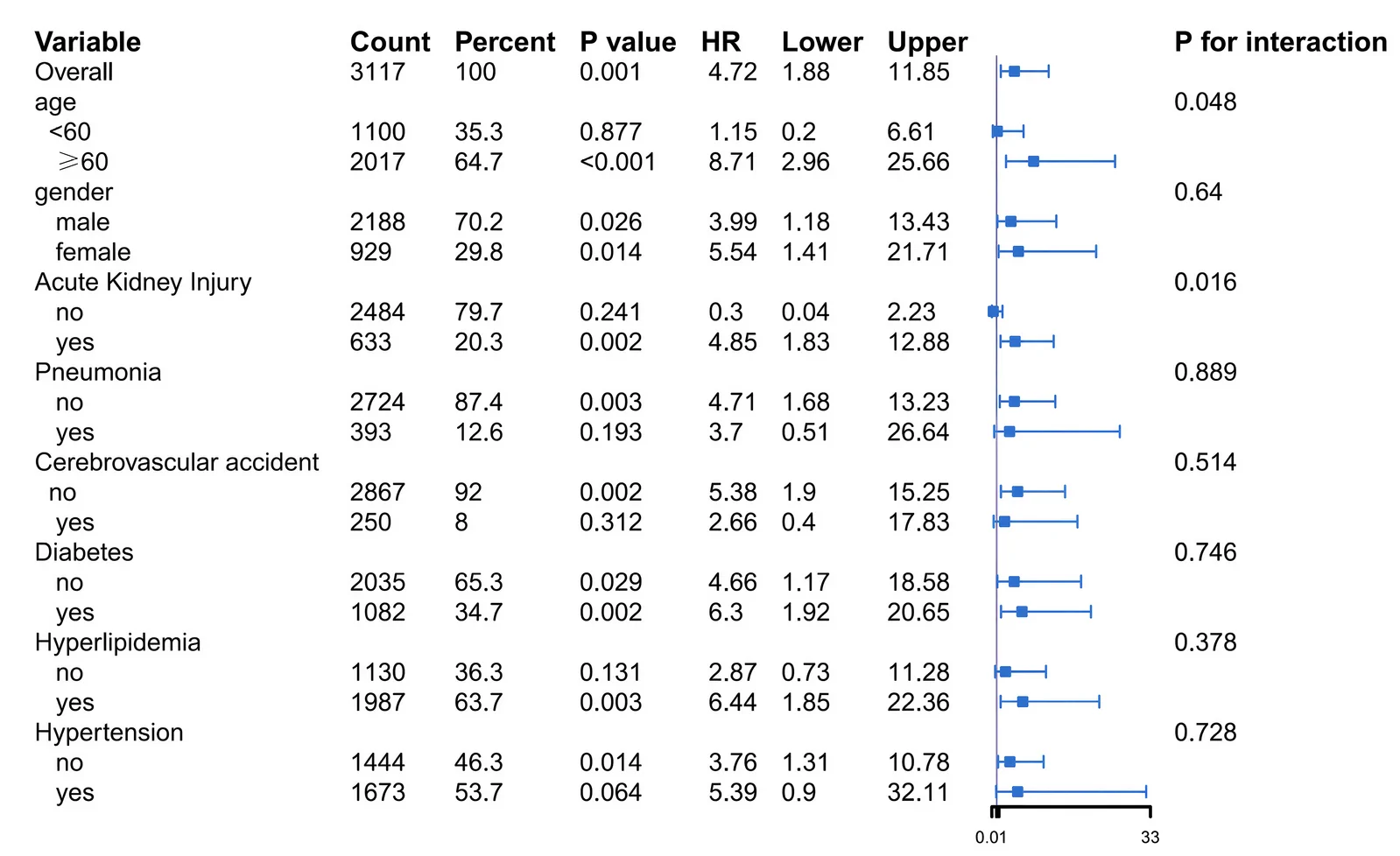
Forest plots of stratified analyses of SHR index and 30-day all-cause mortality. HR = hazard ratio, SHR = stress hyperglycemic ratio.

To validate the association between SHR and 30-day mortality, stratified analyses were performed based on age, gender, cerebrovascular accident, acute kidney injury (AKI), pneumonia, hyperlipidemia, hypertension, and diabetes. Although notable heterogeneity was observed across age strata and between patients with and without AKI, the relationship between SHR and 30-day adverse outcome remained consistent across the other predefined subgroups (Fig. 4). High SHR was significantly associated with an elevated risk of 30-day all-cause mortality specifically within the AKI subgroup (HR: 4.85, 95% confidence interval [CI]: 1.83–12.88) (*P* value for interaction = .016) and the subgroup aged ≥60 years (HR: 8.71, 95% CI: 2.96–25.66) (*P* value for interaction = .048). CS patients with diabetes exhibit higher rates of 30-day mortality (HR: 6.3, 95% CI: 1.92–20.65) compared to those without diabetes (HR: 4.66, 95% CI: 1.17–18.58).

## 4. Discussion

This study demonstrates that SHR serves as an independent predictor of both short- and long-term all-cause mortality in critically ill CS patients. A J-shaped nonlinear dose-response relationship was observed, with escalating mortality risks at higher SHR thresholds. These results suggest that SHR may be a useful adjunctive tool for early risk stratification in intensive care settings, potentially facilitating timely identification of high‑risk patients.

Stress hyperglycemia is a rise in blood glucose that occurs when the body responds to physiological or pathological stress and is associated with the release of hormones such as adrenaline and cortisol, insulin resistance, and an increase in hepatic gluconeogenesis.^[14]^ The 1st blood glucose measurement upon admission is insufficient to effectively assess persistent hyperglycemia, while SHR, as a quantitative assessment marker for stress-induced glycemic derangement, can more accurately reflect blood glucose fluctuation patterns and is significantly associated with disease severity and adverse clinical outcomes.^[8]^ For example, a study by Marenzi et al^[15]^ of 1553 patients with acute myocardial infarction showed that SHR was a more effective predictor of mortality and morbidity during hospitalization than initial admission glucose measurements, and this study further highlights the predictive value of SHR.

In the present study, higher SHR levels were significantly associated with a greater incidence of complications and increased risks of all-cause mortality at 30, 90, 180, and 365 days, consistent with the findings of Cheng et al.^[13]^ This further validates SHR as a critical marker for assessing mortality risk in patients. Previous investigations have demonstrated strong associations between SHR and prognosis in diverse patient populations, including those with aortic stenosis undergoing transcatheter aortic valve replacement^[16]^ and acute coronary syndrome patients undergoing percutaneous coronary intervention. Notably, recent machine-learning models reported a U-shaped relationship between elevated SHR and 360-day all-cause mortality risk in critically ill sepsis patients.^[10]^ In contrast, our study identified a J-shaped association between SHR and all-cause mortality in critically ill CS patients. This discrepancy may be attributed to the study’s prolonged follow-up period, which more comprehensively captures the temporal impact of SHR on mortality, as well as differences in baseline demographics and clinical characteristics of the populations under investigation.

The mechanisms underlying the association between SHR and poor prognosis in CS patients remain incompletely understood, and given the observational nature of our study, the following pathways should be interpreted as speculative and hypothesis-generating rather than causal. Potential mechanisms include stress‑induced immune dysfunction with pro-inflammatory cytokine release,^[17,18]^ osmotic diuresis leading to prerenal insufficiency and impaired wound healing,^[19]^ and endothelial injury mediated by vascular inflammatory activation and reduced nitric oxide bioavailability.^[20]^ Additionally, elevated free fatty acids during stress states may inhibit myocardial glucose utilization, potentially exacerbating ischemia/reperfusion injury.^[21]^

The metabolic memory phenomenon itself triggers persistent pathophysiological alterations even after transient exposure to dysmetabolic conditions, with its clinical impact exhibiting dose- and duration-dependent characteristics.^[22]^ This pathophysiological continuum underscores the critical importance of early glycemic monitoring for SHR management. The metabolic memory phenomenon manifests as irreversible persistence of hyperglycemia-induced damage, wherein prolonged suboptimal glycemic control establishes enduring cellular dysfunction that resists complete reversal even after achieving normoglycemia thereafter. This pathophysiological characteristic crucially underscores the necessity for early identification of high-risk profiles and time-sensitive initiation of evidence-based glycemic regulation strategies, which fundamentally determine long-term therapeutic efficacy in clinical practice.

In clinical practice, where managing critically ill patients remains a persistent challenge, SHR, calculated from routine admission laboratory tests, offers a simple and accessible adjunctive risk-stratification tool. Its use may facilitate early identification of high-risk patients, potentially guiding timely interventions, although this application requires prospective evaluation.

This study has several limitations that warrant consideration. First, the single-center retrospective design inherently constrains the generalizability of findings, necessitating validation through prospective multicenter cohorts to confirm clinical applicability. Second, while our analyses demonstrated a persistent independent association between SHR and all-cause mortality in critically ill CS patients after multivariable adjustment, the observational nature precludes definitive causal inference. Third, data constraints from the MIMIC‑IV database limited access to preoperative laboratory profiles and key intraoperative parameters, such as cardiopulmonary bypass time, aortic cross‑clamp time, surgical complexity, intraoperative bleeding, transfusion requirements, and emergency versus elective surgical status, which may substantially confound mortality risk assessment. Additionally, potential selection bias may arise from the exclusion of cases lacking initial glycemic/HbA1c measurements, though this reflects real-world clinical practice constraints. Finally, formal internal validation, bootstrap, or cross-validation for threshold definition should be conducted in future multicenter external cohort studies.

## 5. Conclusions

The SHR exhibits a nonlinear J-shaped association with all-cause mortality in critically ill CS patients, with elevated levels independently associated with increased mortality risk across short‑ and long‑term follow‑up. These findings suggest that SHR may serve as a useful prognostic discriminator for identifying CS patients. Validation through rigorously designed multicenter prospective studies remains imperative to confirm these findings.

## Acknowledgments

We acknowledged the contributions of the MIMIC-IV (version 3.1) program registry for creating and updating the MIMIC-IV database.

## Author contributions

**Conceptualization:** Yuanli Yang, Jian Xu.

**Formal analysis:** Yuanli Yang.

**Resources:** Ying Zhao.

**Validation:** Ronglin Chen, Linhai Sun.

**Writing – original draft:** Ying Zhao, Jian Xu.

**Writing – review & editing:** Ronglin Chen, Sanming Yu, Linhai Sun.
